# Supplementary material for: Cryptic genetic variation shapes the adaptive evolutionary potential of enzymes
Source: eLife. 2019 Feb 5;8:e40789. doi: 10.7554/eLife.40789 (PMC6372284; doi:10.7554/eLife.40789)
Supplement: Supplementary file 1. — (A) Information about the enzymes characterized in this study. (B). Catalytic parameters of enzymes characterized in this study for PMH and β-lactamase activities. (C) Mutation rate in the NDM1-wt and VIM2-wt naive libraries. (D) Mutations identified in the naive library of VIM2-wt. (E) Mutations identified in the naive library of NDM1-wt. (F) Information on the directed evolution procedure and mutations that were accumulated during directed evolution. (G) PMH fitness values (cell lysate activity) of evolved and designed mutants. (H) Variants identified in the screening of additional round 1 libraries 1–3. (I) Changes in catalytic activity of purified MBL mutants compared to their respective wild-type enzymes, and melting temperature. [file elife-40789-supp1.docx]

*Supplementary file 1 for* Baier at al., Cryptic genetic variation defines the adaptive evolutionary potential of enzymes

**Description of enzymes and directed evolution rounds**

**Supplementary File 1A.** Information about the enzymes characterized in this study.

| **Enzyme name** | **Uniprot ID** | **Genbank accession** | **Organismal source** | **PDB ID code (resolution)** | **References** |
| --- | --- | --- | --- | --- | --- |
| FIM1 | K7SA42 | AFV91534.1 | *Pseudomonas aeruginosa* | n.a. | (1) |
| EBL1 | Q2N9N3 | ABC63608.1 | *Erythrobacter litoralis HTCC 2594* | n.a. | n.a. |
| NDM1 | C7C422 | CAZ39946.1 | *Klebsiella pneumonia* | 3spu (2.1Å) | (2) |
| VIM2 | Q9K2N0 | YP_007509601.1 | *Pseudomonas aeruginosa* | 1ko3 (1.9Å) | (3) |
| VIM1 | Q9XAY4 | CAB46686.1 | *Pseudomonas aeruginosa* | 5n5g (1.3 Å) | (4) |
| VIM7 | Q840P9 | YP_001427370.1 | *Pseudomonas aeruginosa* | 2y87 (1.9Å) | (5) |

n.a, not available

**Supplementary File 1B.** Catalytic parameters of enzymes characterized in this study for PMH and β-lactamase activities.

|  | **PMH Activity** | | | **β-lactamase Activity** | | |
| --- | --- | --- | --- | --- | --- | --- |
| **Enzyme** | *k*_cat_ [s^-1^] | *K*_M_ [μM] | *k*_cat_ *K*_M_ [s^-1^M^-1^] | *k*_cat_ [s^-1^] | *K*_M_ [μM] | *k*_cat_ *K*_M_ [s^-1^M^-1^] |
| Fim1 | **0.0008** ± 0.00005 | **1300** ± 200 | **6.0 × 10^-1^** | **22** ± 1 | **6.5** ± 0.7 | **3.4 × 10^6^** |
| EBL1 | **0.01** ± 0.0003 | **1500** ± 100 | **6.8 × 10^0^** | **13** ± 1 | **6.2** ± 1 | **2.1 × 10^6^** |
| NDM1 | **0.004** ± 0.001 | **13,000** ± 2000 | **3.2 × 10^-1^** | **39** ± 1 | **17** ± 2 | **2.3 × 10^6^** |
| Vim2 | **0.005** ± 0.0001 | **840** ± 50 | **5.9 × 10^0^** | **16** ± 0.7 | **4.2** ± 0.9 | **3.8 × 10^6^** |
| Vim1 | **0.003** ± 0.0005 | **1400** ± 100 | **1.8 × 10^0^** | **170** ± 3 | **100** ± 3 | **1.7 × 10^6^** |
| Vim7 | **0.005** ± 0.0001 | **1900** ± 100 | **2.4 × 10^0^** | **26** ± 0.4 | **9.6** ± 0.6 | **2.7 × 10^6^** |

**Supplementary File 1C.** Mutation rate in the NDM1-wt and VIM2-wt naive libraries.

|  |  | **NDM1** | **VIM2** |
| --- | --- | --- | --- |
|  | **No of variants** | 43 | 44 |
| **Non-synonymous** | **No of mutations** | 93 | 143 |
|  | **mutations / gene** | 2.2 ± 1.4 | 3.3 ± 1.8 |
| **Synonymous** | **No of mutations** | 17 | 44 |
|  | **mutations / gene** | 0.4 ± 0.6 | 1.1 ± 1.1 |
| **Total** | **No of mutations** | 110 | 187 |
|  | **mutations / gene** | 2.6 ± 1.6 | 4.3 ± 2.1 |

**Supplementary File 1D.** Mutations identified in the naive library of VIM2-wt.

|  | **VIM-2** |  |
| --- | --- | --- |
| **Variant** | **Non-synonymous** | **Synonymous** |
| 1 | Y72D, D225G, E242D, G251D, L258W, N264S, N264H, K267P, T270P | p42p, s190s, a224a |
| 2 | D68G, S75F, H122Y, K260Q | g94g |
| 3 | R115C, I235S | g94g, a243a |
| 4 | L86S, V195M, N264D |  |
| 5 | N76S, L194R | l161l |
| 6 | E34G, E45K, V131A, S142P, D223A, Y240S |  |
| 7 | T98P, F121L, S202R | h122h |
| 8 | I54S, D56A, S202R, I210L |  |
| 9 | F121V, H269P | s214s |
| 10 | V38G, Q53P, Q108H, V113G |  |
| 11 | L161P | v80v, y184y |
| 12 | N97D |  |
| 13 | Q108R, S167N, I247T |  |
| 14 | H61Y, I109V, Q238R, H261R |  |
| 15 | D192G |  |
| 16 | E162A, V194L, C208W, N220H, H267R | v58v, p74p, i109i, n271n |
| 17 | W93G, Q238L | t37t, n76n, l148l |
| 18 | Q53R, A141V, V195L, T263A, V265A |  |
| 19 | N97S, T114P, F121L | t37t |
| 20 | Q65R, E106G, I156M, F183C, H189Q | v113v, l258l |
| 21 | Y72D, I79S, E150A, E242A, H269P, N261H | g83g |
| 22 | V43G, L101R, L204R | a243a |
| 23 | Y52S, Q53P, I79M |  |
| 24 | S202G |  |
| 25 |  | a131a |
| 26 | H61Q, V198A | t91t, l132l |
| 27 | S66A, Y184C |  |
| 28 | I109T | d84d, d130d, l194l |
| 29 | I79L, l182R, A187V | g128g, l258l |
| 30 | E40D, D68A, Q108Stop | y35y |
| 31 | S75A, K96Q, R115C, F121L, S214L, L258V |  |
| 32 | L48P, F67S | y35y, l88l |
| 33 | V46D, T262A |  |
| 34 | S144P, Y240D, V266G |  |
| 35 | S160A, V196A | l86l, a141a, a187a |
| 36 | R47W, S75P, V137G, I233V, I236M, Q237P | t37t |
| 37 | E40D, Y52S, G83D, T114P | i41i, a100a |
| 38 | E34A, T37P, I54S | v58v |
| 39 | L86S, L101P, L161P, V246A |  |
| 40 | I156L, F183S, I233S, I236L, T270P, | t37t |
| 41 | K96R, I79M, T158G | a63a |
| 42 | E40G, K267R | y35y, t262t |
| 43 | L161R, Y184S, S202R, Q237H |  |

**Supplementary File 1E.** Mutations identified in the naive library of NDM1-wt

|  | **NDM-1** |  |
| --- | --- | --- |
| **Variant** | **Non-synonymous** | **Synonymous** |
| 1 | G219D, L269R |  |
| 2 | A99T, K206R, N166D |  |
| 3 | I203T |  |
| 4 | no mutation |  |
| 5 | Y140F, M154L, N166T |  |
| 6 | T162P, T201A |  |
| 7 | D212G, K214M, N220S | s63s |
| 8 | M154R | s232s |
| 9 | S75P | a116a |
| 10 | I31T, V89A, V247A, S251F |  |
| 11 | Q60STOP |  |
| 12 | no mutation |  |
| 13 | N57K, L102H, F177V, S249I |  |
| 14 | L102P, N103S, E170G |  |
| 15 | K125E, F163V | t260t |
| 16 | L102P, N193K, L218F, F236L |  |
| 17 | H133R, N220K |  |
| 18 | T226I | n220n |
| 19 | F240S, Stop271C |  |
| 20 | I137T, K242R | f183f |
| 21 | T162A, F236L |  |
| 22 | E40A, F70S, T98A, M265T | a172a |
| 23 | N166H, E170D |  |
| 24 | no mutation |  |
| 25 | no mutation |  |
| 26 | I35M, H102P | t41t |
| 27 | R45R, A157V |  |
| 28 | G42A, N166S, K242E, M245T, K268R |  |
| 29 | M67T, V80A, I210T |  |
| 30 | M39I |  |
| 31 | T201A |  |
| 32 | no mutation |  |
| 33 | D223N |  |
| 34 | I79S, K106R, N142K, K242Q, S251P | l148l |
| 35 | L78V, N146H, I203S | f240f |
| 36 | S63A, L78V, L148P | s251s |
| 37 | H61R, K242M | t41t, v117v, l148l |
| 38 | D48G, D199G, K242Q |  |
| 39 | N57S, F70L | a230a |
| 40 | Y184H |  |
| 41 | D199A, F205C, D254E | d43d |
| 42 | S75P, T162A, F183S, D192N, D202G, I210L |  |
| 43 | N103D | e152e |

**Supplementary File 1F.** Information on the directed evolution procedure and mutations that were accumulated during directed evolution.

| **Round** | **Prescreen^1^** | **NDM1** | **VIM2** | **EBL1** | **VIM7** |
| --- | --- | --- | --- | --- | --- |
| 1 | 4 μg/ml AMP | W93G, N166T | V72A | W93L | W93G, K234E |
| 2 | 4 μg/ml AMP | K211R, G222D (S)^2^ | F67L | M107R, N220H | T232A |
| 3 | 4 μg/ml AMP | Q151R | D223A |  |  |
| 4 | 4 μg/ml AMP | S251F | S202R (S) |  |  |
| 5 | 4 μg/ml AMP | M154V, D96A (S) | G36R, T64A, T263S |  |  |
| 6 | 4 μg/ml AMP | D223E | V41A |  |  |
| 7 | 4 μg/ml AMP | N103K | N154T |  |  |
| 8 | 4 μg/ml AMP | A233V | T191P, V274A |  |  |
| 9 | 250 μM PMH | L49P | E150K, S66P |  |  |
| 10 | 250 μM PMH | V88M | V46D, N264D (S) |  |  |

^1^ pre-screening conditions for either β-lactam antibiotic resistance selection (AMP) or PMH activity screening on agar plates (PMH).

^2^ (S) indicates that DNA Shuffling was performed at this round.

**Supplementary File 1G.** PMH fitness values (cell lysate activity) of evolved and designed mutants.

| **Variant** | **NDM1** | **VIM2** | **EBL1** | **VIM7** |
| --- | --- | --- | --- | --- |
|  | PMH fitness (nM/s) | | | |
| WT | **0.9** ± 0.2 | **3.5** ± 0.1 | **0.1** ± 0.02 | **0.3** ± 0.02 |
| R1 | **34** ± 3 | **7.9** ± 0.3 | **4.3** ± 0.3 | **0.9** ± 0.06 |
| R2 | **190** ± 10 | **13** ± 1 | **31** ± 1 | **1.1** ± 0.1 |
| R3 | **410** ± 20 | **21** ± 1 |  |  |
| R4 | **740** ± 120 | **28** ± 1 |  |  |
| R5 | **1300** ± 150 | **50** ± 1 |  |  |
| R6 | **470** ± 60 | **70** ± 2 |  |  |
| R7 | **910** ± 30 | **70** ± 2 |  |  |
| R8 | **1800** ± 250 | **88** ± 1 |  |  |
| R9 | **1500** ± 300 | **75** ± 1 |  |  |
| R10 | **3300** ± 220 | **120** ± 1 |  |  |
| K211R/G222D | **1.2** ± 0.1 |  |  |  |
| Q151R | **1.1** ± 0.1 |  |  |  |
| S251F | **1.5** ± 0.2 |  |  |  |
| W93G | **20.7** ± 2 | **1.1** ± 0.03 |  |  |
| W93A |  | **1.3** ± 0.07 |  |  |
| W93V |  | **0.72** ± 0.06 |  |  |
| W93L |  | **0.45** ± 0.08 |  |  |
| W93F |  | **1.2** ± 0.09 |  |  |
| F67L |  | **6.5** ± 0.1 |  |  |
| D223A |  | **4.9** ± 0.3 |  |  |
| S202R |  | **6.8** ± 0.3 |  |  |

± indicates standard deviation from triplicate measurements.

The cell lysate was diluted 2-fold prior activity measurement with 500 µM p-nitrophenyl-phenylphosphonate substrate. See Material and Methods for a detailed procedure on PMH fitness measurements.

**Supplementary File 1H.** Variants identified in the screening of additional round 1 libraries 1-3.

| **VIM2 Library** | **Mutations** | **Fitness change in PMH activity^1^** |  |
| --- | --- | --- | --- |
| 1 | D68A, Q108H, P241Q, **T263A (r5)^2^** | 1.4 | |
| 1 | **V72A (r1)** | 1.5 | |
| 1 | **V41G (r6)**, **S66P (r9)**, D68N | 1.4 | |
| 1 | D68A, Q108H, P241Q, **T263A (r5)** | 1.4 | |
| 1 | K107T, **S202G (r4),** E242K | 1.4 | |
| 1 | **V41G (r6)**, **S66P (r9)**, D68N | 1.9 | |
| 2 | **F67L (r2)** | 1.4 | |
| 2 | **F67L (r2)** | 1.6 | |
| 2 | **F67L (r2)** | 1.6 | |
| 2 | **V72A (r1)** | 1.6 | |
| 2 | **F67L (r2)** | 1.4 | |
| 2 | **F67L (r2)** | 1.4 | |
| 2 | Y73F, I79V | 1.1 | |
| 2 | R50Q, **F67L (r2)** | 1.3 | |
| 3 | P157S | 1.3 | |
| 3 | **S202R (r4)** | 1.4 | |
| 3 | V131A | 0.9 | |
| 3 | D84G | 1.0 | |
| 3 | **F67L (r2),** I156T | 1.3 | |
| 3 | A100V, S144T | 1.1 | |
| 3 | D68N | 1.5 | |

| **NDM1 Library** | **Mutations** | **Fitness change in PMH activity^1^** |
| --- | --- | --- |
| 1 | **W93G (r1)**^2^ | 55 |
| 1 | **W93G (r1)** | 45 |
| 1 | **W93G (r1)** | 40 |
| 1 | **W93C (r1)**, K181Q | 25 |
| 1 | **M154T (r5)**, K276R | 2.1 |
| 1 | **M154T (r5)**, K276R | 1.9 |
| 1 | **M154V (r5)** | 1.6 |
| 2 | **W93L (r1)**, N57D | 28 |
| 2 | V113A, H133R | 1.7 |
| 2 | V133A, H133R | 1.5 |
| 2 | V133A, H133R | 1.5 |
| 2 | **M154V (r5)**, D212N | 1.5 |
| 3 | **M154T (r5)** | 2.7 |
| 3 | **M154T (r5)** | 2.3 |
| 3 | **M154T (r5)** | 2.3 |
| 3 | **M154T (r5)** | 2.3 |
| 3 | **M154T (r5)** | 2.2 |
| 3 | **M154T (r5)** | 1.9 |
| 3 | L148F | 1.6 |

^1^ indicates the PMH fitness of the variant relative to that of the wild-type, measured in cell lysate.

^2^ Bolded mutations indicate positions that were also mutated in the original trajectory. The round at which the position was mutated is indicated in between brackets.

**Supplementary File 1I.** Changes in catalytic activity of purified MBL mutants compared to their respective wild-type enzymes, and melting temperature.

| **Enzyme** | **Variant** | **Catalytic activity**  **relative to WT** | | ***T*_m50_ (°C)** |
| --- | --- | --- | --- | --- |
|  |  | PMH | β-lactamase |  |
| **FIM1** | WT | **1.0** ± 0.05 | **1.0** ± 0.05 | **53.5** ± 0.7 |
| **FIM1** | H72V | n.d. | n.d. | **54.0** ± 0.9 |
| **FIM1** | W93G | **6.5** ± 0.30 | **0.9** ± 0.03 | **46.2** ± 1.3 |
| **EBL1** | WT | **1.0** ± 0.05 | **1.0** ± 0.05 | **66.2** ± 0.5 |
| **EBL1** | W93G | **5.0** ± 0.20 | **1.6** ± 0.08 | **64.0** ± 0.8 |
| **EBL1** | P72V | **0.4** ± 0.02 | **1.3** ± 0.06 | **64.8** ± 0.6 |
| **EBL1** | P72A | **0.5** ± 0.02 | **1.8** ± 0.08 | **65.9** ± 0.6 |
| **NDM1** | WT | **1.0** ± 0.09 | **1.0** ± 0.02 | **55.0** ± 0.4 |
| **NDM1** | W93G | **104** ± 7.55 | **2.1** ± 0.05 | **47.4** ± 0.4 |
| **NDM1** | A72V | **0.8** ± 0.15 | **1.3** ± 0.05 | **54.8** ± 0.5 |
| **VIM2** | WT | **1.0** ± 0.01 | **1.0** ± 0.01 | **58.7** ± 0.6 |
| **VIM2** | V72A | **1.7** ± 0.03 | **1.1** ± 0.01 | **57.3** ± 0.6 |
| **VIM2** | W93G | **0.1** ± 0.01 | **0.1** ± 0.01 | **62.0** ± 1.0 |
| **VIM1** | WT | **1.0** ± 0.03 | **1.0** ± 0.01 | **59.0** ± 0.3 |
| **VIM1** | V72A | **3.0** ± 0.15 | **1.1** ± 0.02 | **55.1** ± 0.5 |
| **VIM1** | W93G | **0.3** ± 0.01 | **0.1** ± 0.01 | **55.4** ± 1.8 |
| **VIM7** | WT | **1.0** ± 0.03 | **1.0** ± 0.03 | **56.8** ± 1.0 |
| **VIM7** | V72A | **1.1** ± 0.04 | **0.8** ± 0.05 | **54.1** ± 0.5 |
| **VIM7** | W93G | **3.2** ± 0.14 | **0.2** ± 0.01 | **53.2** ± 0.8 |

n.d, not determined

± indicates standard deviation from triplicate measurement.

All MBL mutants were purified using Strep-tactin affinity chromatography. The catalytic activities of the purified enzymes were measured at single enzyme (5 μM for PMH and 1 nM for β-lactamase activity) and substrate (500 μM for PMH and 100 μM for β-lactamase activity) concentrations.

**Supplementary references**

1. Pollini S, et al. (2013) FIM-1, a new acquired metallo-β-lactamase from a Pseudomonas aeruginosa clinical isolate from Italy. Antimicrobial Agents and Chemotherapy 57(1):410–416.
2. Yong D, et al. (2009) Characterization of a New Metallo-β-Lactamase Gene, *bla*_NDM-1_, and a Novel Erythromycin Esterase Gene Carried on a Unique Genetic Structure in *Klebsiella pneumoniae* Sequence Type 14 from India. Antimicrobial Agents and Chemotherapy 53(12):5046–5054.
3. Bonnin RA, et al. (2013) Complete sequence of broad-host-range plasmid pNOR-2000 harbouring the metallo-β-lactamase gene *bla*_VIM-2_ from *Pseudomonas aeruginosa*. J Antimicrob Chemother 68(5):1060–1065.
4. Lauretti L, et al. (1999) Cloning and characterization of *bla*_VIM_, a new integron-borne metallo-β-lactamase gene from a *Pseudomonas aeruginosa* clinical isolate. Antimicrobial Agents and Chemotherapy 43(7):1584–1590.
5. Li H, Toleman MA, Bennett PM, Jones RN, Walsh TR (2008) Complete Sequence of p07-406, a 24,179-base-pair plasmid harboring the *bla*_VIM-7_ metallo-β-lactamase gene in a *Pseudomonas aeruginosa* isolate from the United States. Antimicrobial Agents and Chemotherapy 52(9):3099–3105.
