# Supplementary material for: Cryptic genetic variation shapes the adaptive evolutionary potential of enzymes
Source: eLife. 2019 Feb 5;8:e40789. doi: 10.7554/eLife.40789 (PMC6372284; doi:10.7554/eLife.40789)
Supplement: Supplementary file 2. — (A) Catalytic parameters of VIM2 variants. (B) Individual catalytic parameters of NDM1 variants. (C) Solubility and melting temperature of NDM1 and Vim2 variants. [file elife-40789-supp2.docx]

*Supplementary file 2 for* Baier at al., Cryptic genetic variation defines the adaptive evolutionary potential of enzymes

**Kinetic parameters of all variants**

**Supplementary File 2A.** Catalytic parameters of VIM2 variants.

|  | **PMH activity** | | | **β-lactamase activity** | | |
| --- | --- | --- | --- | --- | --- | --- |
| **Variant** | *k*_cat_ [s^-1^] | *K*_M_ [μM] | *k*_cat_ *K*_M_ [s^-1^M^-1^] | *k*_cat_ [s^-1^] | *K*_M_ [μM] | *k*_cat_ *K*_M_ [s^-1^M^-1^] |
| WT | **0.005** ± 0.0001 | **840** ± 50 | **5.9** × **10^0^** | **16** ± 0.7 | **4.2** ± 0.9 | **3.8** × **10^6^** |
| R1 | **0.03** ± 0.001 | **2100**± 110 | **1.3** × **10^1^** | **17** ± 1 | **4.7** ± 1.3 | **3.7** × **10^6^** |
| R2 | **0.02** ± 0.001 | **1500** ± 100 | **1.6** × **10^1^** | **23** ± 1 | **9.6** ± 2.3 | **2.4** × **10^6^** |
| R3 | **0.04** ± 0.0005 | **850** ± 30 | **4.8** × **10^1^** | **37** ± 2 | **100** ± 21 | **3.7** × **10^5^** |
| R4 | **0.07** ± 0.001 | **1200** ± 100 | **5.8** × **10^1^** | **32** ± 3 | **21** ± 6.1 | **1.5** × **10^6^** |
| R5 | **0.09** ± 0.002 | **640** ± 30 | **1.4** × **10^2^** | **53** ± 1 | **37** ± 3.5 | **1.4** × **10^6^** |
| R6 | **0.15** ± 0.004 | **880** ± 70 | **1.8** × **10^2^** | **40** ± 6 | **110** ± 46 | **3.7** × **10^5^** |
| R7 | **0.08** ± 0.003 | **890** ± 90 | **9.3** × **10^1^** | **17** ± 2 | **32** ± 11 | **5.4** × **10^5^** |
| R8 | **0.15** ± 0.01 | **890** ± 80 | **1.6** × **10^2^** | **33** ± 4 | **55** ± 19 | **6.0** × **10^5^** |
| R9 | **0.15** ± 0.007 | **1300** ± 200 | **1.2** × **10^2^** | **19** ± 0.3 | **33** ± 3 | **5.8** × **10^5^** |
| R10 | **0.15** ± 0.005 | **1000** ± 100 | **1.4** × **10^2^** | **15** ± 0.3 | **34** ± 3 | **4.6** × **10^5^** |

± Indicates the error in the fit of the data to the Michaelis Menten equation.

**Supplementary File 2B.** Individual catalytic parameters of NDM1 variants.

|  | | **PMH activity** | | | **β-lactamase activity** | | |
| --- | --- | --- | --- | --- | --- | --- | --- |
| **Variant** | *k*_cat_ (s^-1^) | | *K*_M (_μM] | *k*_cat_ *K*_M_ [s^-1^M^-1^] | *k*_cat_ [s^-1^] | *K*_M_ [μM] | *k*_cat_ *K*_M_ [s^-1^M^-1^] |
| WT | **0.004** ± 0.001 | | **13,000** ± 2000 | **3.2** ×**10^-1^** | **39** ± 1 | **17** ± 2 | **2.3** × **10^6^** |
| R1 | **0.5** ± 0.02 | | **5100** ± 400 | **1.0** ×**10^2^** | **63** ± 1 | **30** ± 2 | **2.1** × **10^6^** |
| R2 | **1.2** ± 0.1 | | **6500** ± 700 | **1.8** ×**10^2^** | **140** ± 5 | **60** ± 7 | **2.3** × **10^6^** |
| R3 | **0.9** ± 0.02 | | **4500** ± 300 | **2.0** ×**10^2^** | **87** ± 2 | **70** ± 6 | **1.3** × **10^6^** |
| R4 | **1.8** ± 0.02 | | **3300** ± 100 | **5.5** ×**10^2^** | **57** ± 1 | **32** ± 3 | **1.8** × **10^6^** |
| R5 | **2.1** ± 0.1 | | **2400** ± 100 | **9.1 ×10^2^** | **54** ± 2 | **20** ± 4 | **2.8** × **10^6^** |
| R6 | **18** ± 0.6 | | **8500** ± 400 | **2.1** ×**10^3^** | n.d. | n.d. | n.d. |
| R7 | **11** ± 0.1 | | **1600** ± 170 | **7.2** ×**10^3^** | **120** ± 3 | **65** ± 6 | **1.9** × **10^6^** |
| R8 | **15** ± 0.1 | | **1700** ± 160 | **8.7** ×**10^3^** | **96** ± 2 | **61** ± 4 | **2.4** × **10^6^** |
| R9 | **7** ± 0.1 | | **1400** ± 300 | **5.1** ×**10^3^** | **115** ± 3 | **63** ± 5 | **1.8** × **10^6^** |
| R10 | **8** ± 0.4 | | **2000** ± 130 | **5.9** ×**10^3^** | **103** ± 6 | **48** ± 6 | **2.1** × **10^6^** |

n.d, not determined

± Indicates the error in the fit of the data to the Michaelis Menten equation.

**Supplementary File 2C.** Solubility and melting temperature of NDM1 and VIM2 variants.

|  | **NDM1** | | **VIM2** | |
| --- | --- | --- | --- | --- |
| **Variant** | Solubility (%) | *T*_m_ (°C) | Solubility (%) | *T*_m_ (°C) |
| WT | 43 | **55.0** ± 0.4 | 40 | **58.7** ± 0.6 |
| R1 | 25 | **48.1** ± 0.6 | 44 | **57.3** ± 0.6 |
| R2 | 21 | **51.0** ± 0.5 | 42 | **60.3** ± 2.1 |
| R3 | 27 | **52.4** ± 0.8 | 37 | **56.3** ± 1.5 |
| R4 | 16 | **53.1** ± 1.1 | 48 | **58.0** ± 0.1 |
| R5 | 18 | **51.9** ± 0.3 | 57 | **54.3** ± 2.3 |
| R6 | 18 | **50.1** ± 0.8 | 62 | **54.3** ± 2.3 |
| R7 | 21 | **51.0** ± 0.7 | 61 | **55.3** ± 1.2 |
| R8 | 28 | **55.8** ± 0.8 | 69 | **61.7** ± 0.6 |
| R9 | 16 | **54.1** ± 0.4 | 86 | **55.3** ± 0.6 |
| R10 | 34 | **55.0** ± 0.9 | 67 | **55.3** ± 0.6 |

± indicates standard deviation from triplicate measurements.
