# Supplementary material for: Cryptic genetic variation shapes the adaptive evolutionary potential of enzymes
Source: eLife. 2019 Feb 5;8:e40789. doi: 10.7554/eLife.40789 (PMC6372284; doi:10.7554/eLife.40789)
Supplement: Supplementary file 3. — Crystallographic data collection and refinement statistics. [file elife-40789-supp3.docx]

*Supplementary file 3 for* Baier at al., Cryptic genetic variation defines the adaptive evolutionary potential of enzymes

**Crystallographic information**

**Supplementary File 3**. Crystallographic data collection and refinement statistics.

| Variant | **NDM1-R10** | **NDM1-R10** (product) | **VIM2-R10** |
| --- | --- | --- | --- |
| PDB ID | 5JQJ | 5K4M | 6BM9 |
| Wavelength (Å) | 0.9537 | 0.9537 | 0.9537 |
| No. copies in an asymmetric unit | 1 | 1 | 4 |
| Resolution range (Å) | 34.45-1.67 | 39.13-1.98 | 37.89-2.19 |
|  | (1.73-1.67) | (2.05-1.98) | (2.27-2.19) |
| Space group | C 2 2 2_1_ | C 2 2 2_1_ | C 1 2 1 |
| Unit cell (Å, °) | 37.80 137.72 77.46 90 90 90 | 37.82 138.16 78.20 90 90 90 | 128.60 41.67 156.76  90 99 90 |
| Total reflections | 46608 (3668) | 29257 (2838) | 150766 (13607) |
| Unique reflections | 23318 (1837) | 14613 (1421) | 41963 (4025) |
| Multiplicity | 2.0 (2.0) | 2.0 (2.0) | 3.6 (3.4) |
| Completeness (%) | 97.22 (77.72) | 99.72 (99.37) | 97.82 (96.08) |
| Mean I/sigma(I) | 17.93 (1.89) | 11.13 (5.78) | 11.28 (1.86) |
| Wilson B-factor (Å^2^) | 19.27 | 14.37 | 32.54 |
| ^a^R-merge | 0.029 (0.46) | 0.029 (0.08) | 0.094 (0.76) |
| ^b^R-meas | 0.04 | 0.04 | 0.11 |
| ^c^CC1/2 | 0.999 (0.588) | 0.999 (0.978) | 0.996 (0.609) |
| ^d^CC* | 1.00 (0.861) | 1.00 (0.994) | 0.999 (0.870) |
| R-work | 0.144 (0.240) | 0.142 (0.150) | 0.223 (0.323) |
| R-free | 0.187 (0.287) | 0.208 (0.198) | 0.268 (0.337) |
| Number of non-hydrogen atoms | 2052 | 1982 | 7053 |
| macromolecules | 1758 | 1749 | 6842 |
| ligands | 28 | 46 | 16 |
| water | 266 | 187 | 195 |
| Protein residues | 230 | 230 | 902 |
| RMS(bonds) (Å) | 0.02 | 0.017 | 0.003 |
| RMS(angles) (°) | 2.08 | 1.95 | 0.76 |
| Ramachandran preferred (%) | 98.05 | 96.63 | 95.71 |
| Ramachandran allowed (%) | 0.98 | 2.4 | 3.50 |
| Ramachandran outliers (%) | 0.98 | 0.96 | 0.79 |
| Clashscore | 3.95 | 5.67 | 4.94 |
| Average B-factor (Å^2^) | 24 | 16.8 | 45.1 |
| macromolecules | 22.1 | 15.4 | 45.2 |
| ligands | 36.8 | 29.7 | 57.3 |
| solvent | 35 | 26.6 | 40.9 |

* Highest-resolution shell is shown in parentheses.

^a^ R-merge $=$ $\sum_{hkl} \sum_{i} \left| I_{i}\left( hkl \right)- \left\langle I\left( hkl \right) \right\rangle\right|/\sum_{hkl} \sum_{i} I_{i}(hkl)$^85^.

^b^ R-meas = ${\sum_{hkl} \{N(hkl)/[N(hkl)-1]\}}^{1/2}$ $\times\sum_{i} |I_{i}(hkl)-\left\langle I\left( hkl \right) \right\rangle|\sum_{i} I_{i}(hkl)$^86^.

^c^ CC1/2$= \sum(x-\left\langle x \right\rangle)(y-\left\langle y \right\rangle)/\left[ {\sum(x-\left\langle x \right\rangle)}^{2}\sum{(y-\left\langle y \right\rangle)}^{2} \right]^{1/2}$^65,87^.

^d^ CC* $= \sqrt{\frac{{2CC}_{1/2}}{1 +{CC}_{1/2}}}$^65,87^.
