# Supplementary material for: Cryptic genetic variation shapes the adaptive evolutionary potential of enzymes
Source: eLife. 2019 Feb 5;8:e40789. doi: 10.7554/eLife.40789 (PMC6372284; doi:10.7554/eLife.40789)
Supplement: Supplementary file 4. — (A) Parameters used to describe the Zn2+ ions in our MD simulations. (B) List of relevant ionized states as well as the protonation patterns of histidine residues in our molecular dynamics simulations. All other residues were kept in their unionized forms as they were outside the simulation sphere (see main text). [file elife-40789-supp4.docx]

*Supplementary file 4 for* Baier at al., Cryptic genetic variation defines the adaptive evolutionary potential of enzymes

**Molecular dynamics information**

**Supplementary File 4A**. Parameters used to describe the Zn^2+^ ions in our MD simulations.

**
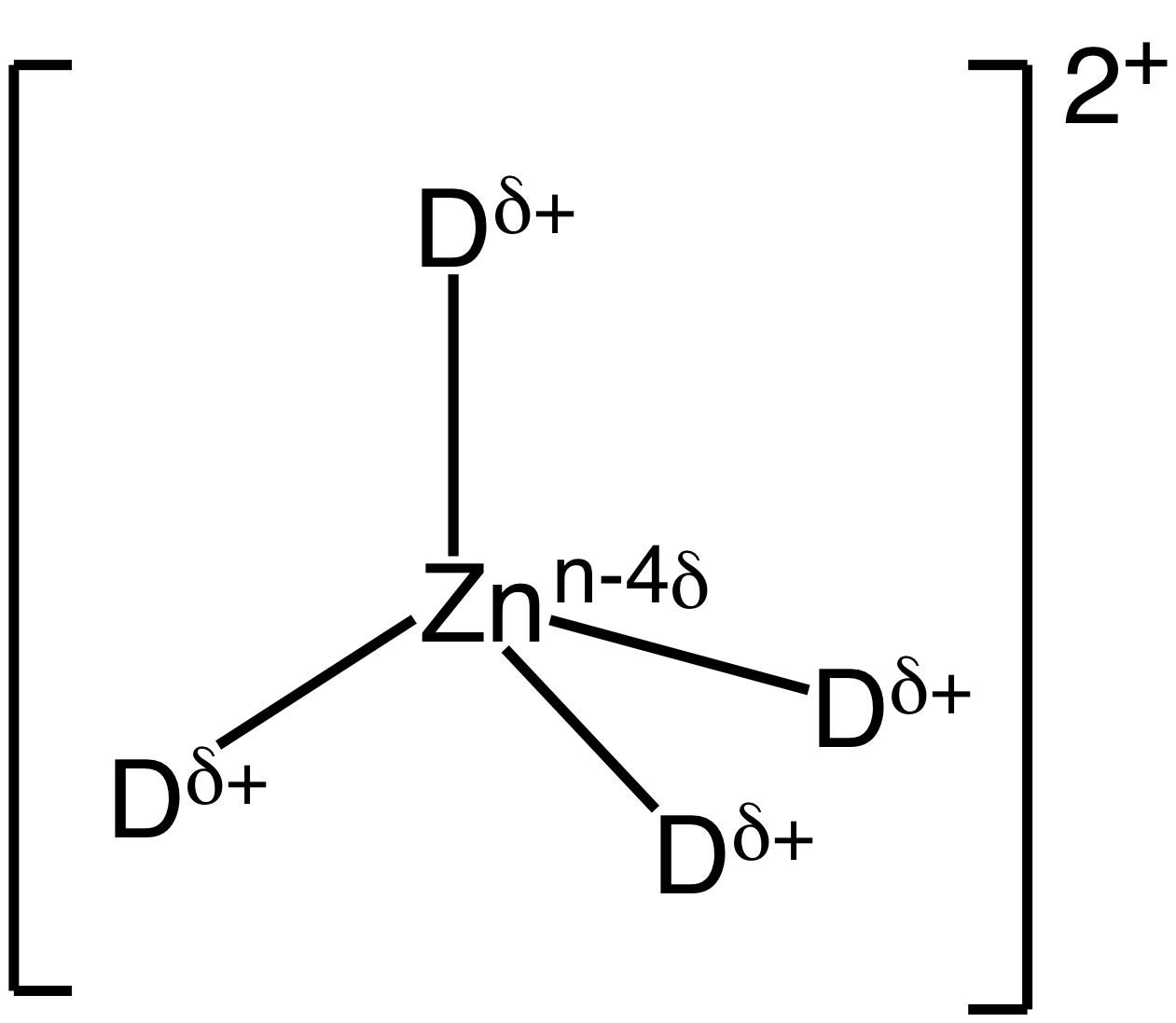
**
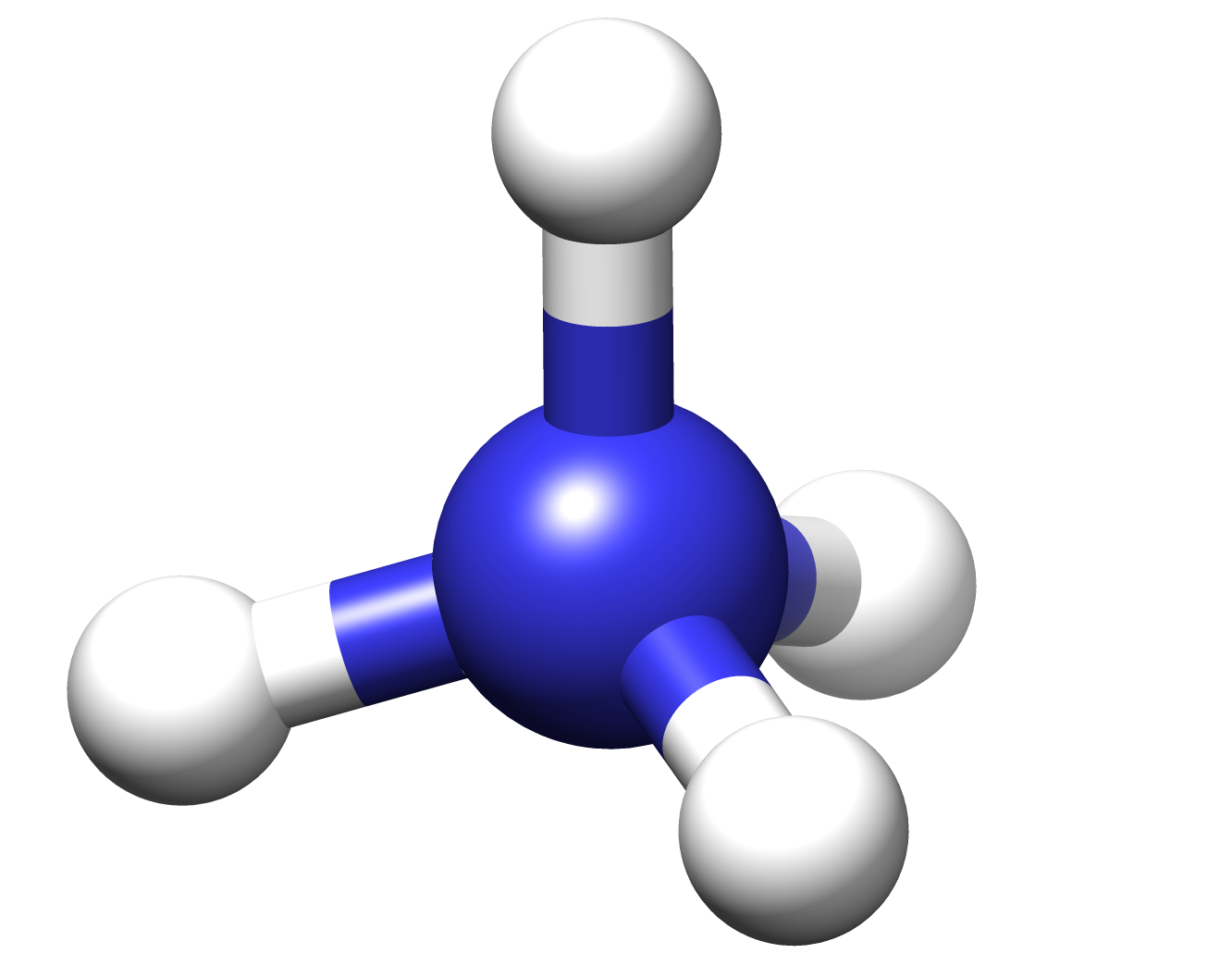


| **bond type^a^** | ***K*_b_** | | | ***r*_0_** | | |
| --- | --- | --- | --- | --- | --- | --- |
| Zn-D | 800.0 | | | 0.90 | | |
| D-D | 800.0 | | | 1.47 | | |
| **angle type^b^** | ***K*_θ_** | | | ***θ*_0_** | | |
| D-Zn-D | 250.0 | | | 109.50 | | |
| D-D-Zn | 250.0 | | | 32.25 | | |
| D-D-D | 250.0 | | | 60.00 | | |
| **atom type** | **mass** | **charge** | ***A*_i_^c^** | | ***B*_i_^c^** |  |
| Zn | 53.4 | 0.00^d^/-1.00^e^ | 41.00 | | 32.00 |  |
| D | 3.0 | 0.50^d^/0.75^e^ | 0.05 | | 0.05^d^/0.00^e^ |  |

^a^ *U*_b_ = *K*_b_(*b* – *b*_0_)^2^, where *K*_b_ is in kcal mol^-1^Å^-2^ and *r*_0_ is in Å. ^b^ *U_θ_* =  (1/2)*k*_θ_ (*θ* − *θ*_0_)^2^ where *K*_θ_ is in kcal mol^−1^ rad^−2^ and *θ*_0_ is in degrees. ^c^ Lennard-Jones parameters given in units of [kcal^1/2^ mol^−1/2^ Å^−6^] for *A*_i_ and [kcal^1/2^ mol^−1/2^ Å^−3^] for *B*_i_. ^d^ Parameters used for the metal ion coordinated by His120, His122, His189 (NDM1 numbering) ^e^Parameters used for the metal ion coordinated by Asp124, Cys208, His250 (NDM1 numbering).

**Supplementary File 4B.** List of relevant ionized states as well as the protonation patterns of histidine residues in our molecular dynamics simulations. All other residues were kept in their unionized forms as they were outside the simulation sphere (see main text).

| Residue | Residue number | | |
| --- | --- | --- | --- |
|  | NDM1 WT and W93G | NDM1 R10 | VIM2 |
| Asp | 66, 90, 95, 124, 130, 192, 212, 223, 225, 254 | 66, 90, 95, 124, 130, 192, 212, 222, 225, 254 | 62, 84, 119, 120, 126, 199, 236, 238 |
| Glu | 152 | 152, 223 | 147, 149, 166, 225 |
| Lys | 125, 211, 216 | 125, 216 | 90 |
| Arg | - | 151, 211 | 121, 129, 143, 144, 228, 296 |
| His-δ | 250, 133, 120, 189 | 250, 133, 120, 189 | 116,196 263 |
| His-ε | 61, 122, 159 | 61, 122, 159 | 55, 118, 170, 252, 285, 293 |
| Hip | 228, 261 | 228, 261 | - |
